# The Influence of Non-Thermal Plasma Treatment on Osseointegration of Endosteal Implants Presenting Decompressing Vertical Chambers

**DOI:** 10.3390/bioengineering13040472

**Published:** 2026-04-17

**Authors:** Shray Mehra, Hana Shah, Sara E. Munkwitz, Nicholas J. Iglesias, Tina Joshua, Kashyap K. Tadisina, Natalia Fullerton, Vasudev Vivekanand Nayak, Lukasz Witek, Paulo G. Coelho

**Affiliations:** 1 University of Miami Miller School of Medicine, Miami, FL 33136, USA; 2 Florida International University Herbert Wertheim College of Medicine, Miami, FL 33199, USA; 3 DeWitt Daughtry Family Department of Surgery, University of Miami Miller School of Medicine, Miami, FL 33136, USA; 4 William Carey University College of Osteopathic Medicine, Hattiesburg, MS 39401, USA; 5 DeWitt Daughtry Family Department of Surgery, Division of Plastic Surgery, University of Miami Miller School of Medicine, Miami, FL 33136, USA; 6 Department of Biochemistry and Molecular Biology, University of Miami Miller School of Medicine, Miami, FL 33136, USA; 7 Dr. John T. Macdonald Foundation Biomedical Nanotechnology Institute, University of Miami, Miami, FL 33136, USA; 8 Biomaterials and Regenerative Biology Division, NYU College of Dentistry, New York, NY 10010, USA; 9 Department of Biomedical Engineering, NYU Tandon School of Engineering, Brooklyn, NY 11201, USA; 10 Department of Oral and Maxillofacial Surgery, NYU College of Dentistry, New York, NY 10010, USA; 11 Hansjörg Wyss Department of Plastic Surgery, NYU Grossman School of Medicine, New York, NY 10016, USA; 12 Sylvester Comprehensive Cancer Center, University of Miami Miller School of Medicine, Miami, FL 33136, USA

**Keywords:** endosteal implants, osseointegration, thread design, implant geometry, histomorphometry

## Abstract

Current evidence suggests that achieving the desired level of osseointegration necessitates a hierarchical approach to implant design. This is particularly relevant for osseointegration around implant systems such as those presenting vertical decompression chambers and acid-etched surfaces which could further be augmented by non-thermal plasma (NTP) treatment. Three implant systems were compared in this study: (i) ND (GM Helix Acqua Implant; Neodent^®^, Curitiba, PR, Brazil—hybrid, acid-etched thread design treated with isotonic sodium chloride solution), (ii) Sin (Epikut Plus; S.I.N. Implant System, São Paulo, Brazil—V-shaped, acid-etched thread design treated with nano-hydroxyapatite), and (iii) Mp (Maestro; Implacil De Bortoli, São Paulo, Brazil—buttress, acid-etched thread design with decompressing vertical chambers). The ND and Sin implants were used directly as supplied by the manufacturer. For the Mp implants, the manufacturer-supplied surface was subjected to supplemental acid etching with 37% hydrochloric acid followed by Argon-based NTP treatment administered with a pulsed plasma generator prior to implantation into the iliac crest of n = 12 adult female sheep. Histomorphometric analysis was conducted at 3- and 12-week post-implantation (n = 6 sheep per time point) to assess bone-to-implant contact (BIC) and bone area fraction occupancy (BAFO). After 3 weeks in vivo, the healing chambers of all implant groups consisted predominantly of newly forming woven bone. By 12 weeks, bone maturation was observed, with the presence of remodeling sites and some areas of well-organized lamellar structures occupying the healing chambers. At both 3 and 12 weeks, the Mp implants demonstrated significantly higher BAFO values relative to ND (*p* = 0.015 and *p* = 0.008, respectively). The combination of vertical healing chambers, acid etching, and NTP treatment promoted early vascular infiltration and sustained bone deposition.

## 1. Introduction

Endosteal implants are widely used in dental and craniofacial reconstruction, yet it has been reported that over 40% of implants fail due to excessive mobility, necessitating improvement of the implant–bone interface for short- and long-term stability [1]. Efforts have been directed towards hierarchical implant design at the macro-, micro-, and nano-geometric length scales. On a macrogeometric scale, conical implants are reported to provide superior stability relative to cylindrical counterparts [2,3]. The conical implant enables easier insertion and greater stability owing to vertical and lateral bone contact [2]. With this design, compression enhances mechanical engagement and assists in more favorable load transmission on bone [2,4,5]. On the other hand, cylindrical implants have a uniform diameter from the coronal to the apical aspect of the implant body and rely on frictional forces between the implant surface and bone walls for stability. Consequently, they depend on a precise fit at the osteotomy [2]. To combine the mechanical advantages of both designs, hybrid implants, which feature a narrow and cylindrical-shaped coronal section and a tapered apical portion, have been developed. For example, in a pilot study by Nevins et al., adequate primary and secondary stability was observed with a hybrid implant design [6].

Thread design also plays a critical role in bone-implant contact and stability [7]. Threads increase the surface area available for osseointegration and help distribute functional load along the implant–bone interface [3]. Typically, implants with V-shaped threads have demonstrated greater stability compared to square threads due to their ability to generate compressive stress [2]. Building on this principle, more recent designs have introduced vertical decompression chambers, which further enhance bone-implant contact relative to conventional thread designs [8]. These vertical healing chambers create healing spaces that support intramembranous bone formation [9,10]. 

Beyond macrogeometry, microgeometry also plays a crucial role in enhancing biological response at the implant–bone interface. Surface characteristics at this reduced length scale can be modified via chemical and physical methods like grit blasting and/or acid etching. In grit blasting, the outer surface of an implant is impinged with abrasive particles to alter the surface texture [11]. One common method uses alumina or titanium oxide particles to induce surface roughness values between 0.7 and 1.2 µm—a range reported to promote osteoblast activity [12,13]. Similarly, acid etching involves corrosion of the implant surface to create micro pits [13,14]. Beyond acid etching and grit blasting, a variety of surface coatings have been developed to increase hydrophilicity and surface wettability [15]. Hydroxyapatite (HA) coating is one such technique which renders surfaces hydrophilic and mimics the inorganic matrix of bone by serving as a reservoir of calcium and phosphate ions to promote osteoblast proliferation and bone matrix deposition [16,17,18,19]. Similarly, surface treatment with isotonic 0.9% sodium chloride (NaCl) solution has been theorized to hydroxylate the titanium oxide layer to increase surface reactivity and attract positively charged molecules, like plasma proteins and growth factors, to improve intramembranous bone formation [20,21,22].

More recently, however, non-thermal plasma (NTP) treatment has emerged as a promising surface treatment technique [23]. NTP uses a partially ionized gas that operates in thermodynamic non-equilibrium, in which electrons possess high energies while the bulk gas remains at ambient temperatures [24]. NTP can be generated using dielectric barrier discharges and plasma jets [24,25,26]. Depending on the working gas and operating conditions, NTP can produce reactive oxygen and nitrogen species (RONS) and UV photons, among others [24,25,26]. Substrate surface temperatures during NTP exposure remain below 40 °C, supporting its non-thermal nature and suitability for thermally sensitive biomaterials [27,28]. The underlying mechanism of NTP relies on increasing the surface energy of the implant by removing hydrocarbon contamination [29,30,31]. This has been shown in previous studies via X-ray photoelectron spectroscopy and sessile drop tests [28,32,33]. Without altering the bulk chemical composition of the implant, NTP has been demonstrated to improve protein adsorption and accelerate early bone formation [30,34].

Recent findings suggest that a combination of macrogeometry and surface physicochemical modifications may exert a synergistic effect, improving the biological interface between implant surfaces and osteotomy walls [35]. While individual implant design parameters have been assessed separately, current evidence indicates that desired levels of osseointegration are unlikely to be achieved by altering a single factor; thus, hierarchical design of dental implants is required [9]. This is particularly relevant for osseointegration around newer implant systems, such as those presenting vertical decompression chambers and acid-etched surfaces, that could further be augmented by NTP application [36]. Therefore, the aim of this study was to histologically and histomorphometrically assess the effect of implant design features spanning macro- to nanometer length scales on osseointegration in a challenging, type IV bone environment.

## 2. Materials and Methods

### 2.1. Implants

**Figure 1:**
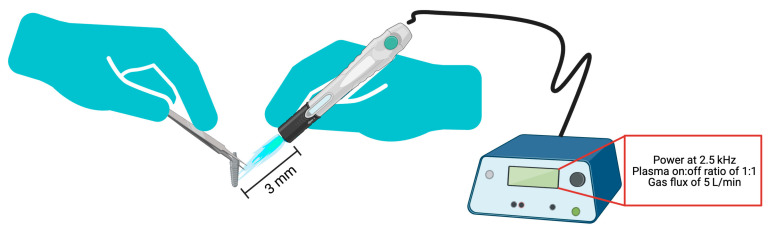
Schematic of the NTP treatment. NTP was applied for 60 seconds (s) at a distance of ~3 mm from the implant surface. The treated implants were subsequently placed into the osteotomy sites in the sheep illium. Schematic generated on biorender.com.

Three commercially available Titanium (Ti) implant systems were compared in this study: (1) ND (GM Helix Acqua Implant; Neodent^®^, Paraná, Brazil) [37], (2) Sin (Epikut Plus; S.I.N. Implant System, São Paulo, Brazil) [38], and (3) Mp (Maestro; Implacil De Bortoli, São Paulo, Brazil) [39]. A summary of the implant features is provided in Table 1. The ND and Sin implants were used directly as supplied by the manufacturer. For the Mp implants, the manufacturer-supplied surface (blasted with titanium oxide microparticles (~100 μm) and subsequently etched with maleic acid), was subjected to supplemental acid etching using our proprietary 37% hydrochloric acid (HCl) etching protocol, as previously described [40]. The dual acid-etched Mp implants were then subjected to Argon-based NTP which was administered with a pulsed plasma generator (KinPen, INP-Greifswald, Greifswald, Germany) operating at 2.5 kHz with a plasma on:off ratio of 1:1 and at a gas flux of 5 L/min supplied via an integrated gas flow control system. The outlet nozzle of the NTP device was held ~3 mm from the implant surface, and a gentle sweeping motion was used to pass the NTP plume over the implant. This was to ensure that the plume coated the healing chambers prior to implantation, as per our previous work [41] (Figure 1).

### 2.2. Surgical Procedure

This study received approval from the Institutional Animal Care and Use Committee of École Nationale Vétérinaire d’Alfort (ENVA, Maisons-Alfort, Ile-de-France, France, file number: 13-011; notice number: 05/14/13-3). The in vivo experiments were completed in compliance with Animal Research Reporting of In Vivo Experiments guidelines. N = 12 female sheep, each weighing ~65 kg, were procured and allowed to acclimate at the ENVA facility for 1 week prior to surgical procedures. The iliac crest was selected as the site of implantation due to its predominantly trabecular architecture that mimics the bone quality and type typically seen in the posterior maxilla in clinical settings. All procedures were conducted under general anesthesia, and induction was performed with sodium pentothal (15–20 mg/kg IV in Normasol solution) via the jugular vein. Anesthesia was maintained with inhaled isoflurane (1.5–3%) in a 50/50 O_2_/N_2_O mixture. Throughout the surgery, animals were monitored using electrocardiography, waveform capnography, and pulse oximetry. Body temperature was regulated using a circulating warm water blanket and was actively monitored throughout the surgery. Thereafter, the iliac crest was shaved and prepared aseptically with iodine solution.

A ~10 cm skin incision was made, followed by dissection of subcutaneous fat and muscle layers. A periosteal elevator was used to expose the bone, and the osteotomy sites were prepared using sequential drilling protocols as recommended by the respective implant manufacturers [37,38,39]. Each sheep received 3 implants [1 of each type (ND, Sin, and Mp)] in a randomized fashion to reduce site bias. Cefazolin (500 mg) was administered pre-operatively and post-operatively for infection prophylaxis. Post-operatively, animals were provided with food and water ad libitum. Sheep were euthanized by anesthetic overdose following institutional ethical guidelines at 3 or 12 weeks post-operatively (n = 6 sheep per time point). Upon euthanasia, the iliac samples (including the implant and surrounding bone) were harvested en bloc via sharp dissection, and processed for histological and histomorphometric evaluation.

### 2.3. Histological Processing and Analysis

The samples underwent fixation in 10% formalin for 24 hours (h) followed by sequential dehydration in ethanol (70–100% *v*/*v*) for 96 h. Subsequently, samples were immersed in methyl salicylate for 48 h and embedded in a methacrylate-based resin. After polymerization of the methacrylate resin, the samples were subjected to UV crosslinking for 72 h. Sections (~300 µm thick) were obtained along the longitudinal-axis of the implants using a low-speed precision wafering saw (Isomet 2000, Buehler Ltd., Lake Bluff, IL, USA). The sections were glued to acrylic slides using a low-viscosity cyanoacrylate adhesive (Loctite 408, Henkel AG & Co. KGaA, Düsseldorf, Germany). After setting for ~12 h, the slides were then ground using silicon carbide abrasive sheets of progressively finer grit sizes (400, 600, 800, 1200 grit) under continuous irrigation with water until a final thickness of ~100 µm was achieved. Slides were polished using a microfiber cloth coated with an alumina-based polishing suspension (MicroPolish^TM^ (1 μm), Buehler, Lake Bluff, IL, USA). The sections were then stained with Stevenel’s Blue and Van Gieson’s Picro Fuchsin (SVG) and high-resolution images were obtained using an automated slide scanner with specialized software (Aperio CS2 and Imagescope v12.4.6, Leica Biosystems, Deer Park, IL, USA). Bone-to-implant contact (BIC) and bone area fraction occupancy (BAFO) were quantified to evaluate the degree of osseointegration around the implants, expressed as percentages, as shown previously [43]. All analyses were performed by a single trained investigator who was blinded to the implant group assignments.

### 2.4. Statistical Analysis

Data normality was assessed using Shapiro–Wilk tests (*p* > 0.05), after which statistical analysis was performed using a mixed model analysis followed by least significant difference (LSD) post hoc analysis with fixed factors of time and implant. This analysis was performed to due to nested within subject observations. Statistical analysis was performed on SPSS (v31, IBM Corp., Armonk, NY, USA). All values were reported as mean ± 95% confidence intervals (95%CI), unless otherwise specified. A *p*-value <0.05 was considered statistically significant.

## 3. Results

**Figure 2:**
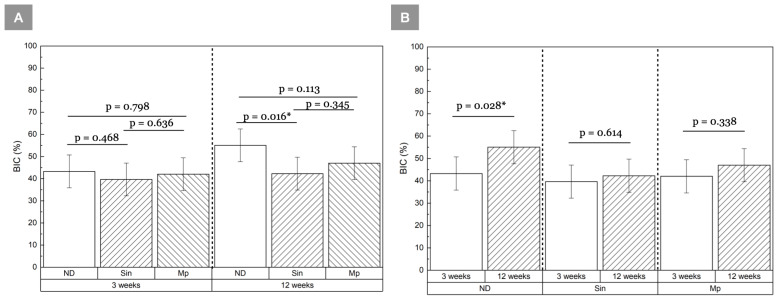
BIC (%) compared (**A**) among groups at 3 and 12 week time points, and (**B**) as a function of time for each implant group. Results shown as mean ± 95% CI, * indicates *p* < 0.05.

**Figure 3:**
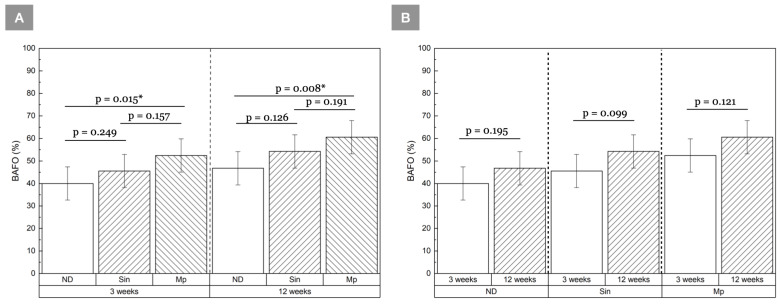
BAFO (%) compared (**A**) among groups at 3 and 12 week time points, and (**B**) as a function of time for each implant group. Results shown as mean ± 95% CI, * indicates *p* < 0.05.

**Figure 4:**
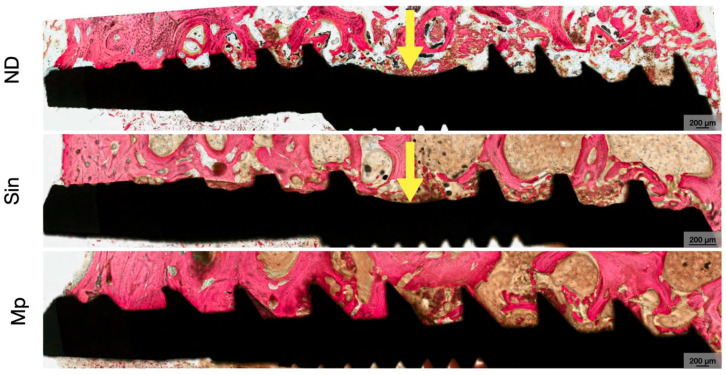
Representative low magnification histomicrographs of ND, Sin, and Mp implants at 3 weeks. Implants are shown in black and bone is in red as a result of SVG staining, with cutting threads (in ND and Sin) demarcated with yellow arrows.

**Figure 5:**
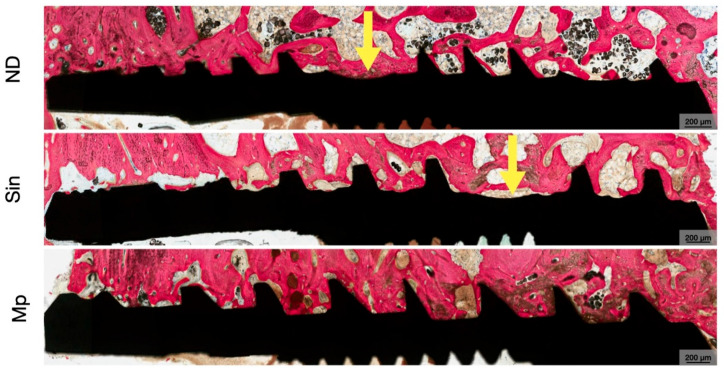
Representative low magnification histomicrographs of ND, Sin, and Mp implants at 12 weeks. Implants are shown in black, and bone is in red as a result of SVG staining, with cutting threads (in ND and Sin) demarcated with yellow arrows.

**Figure 6:**
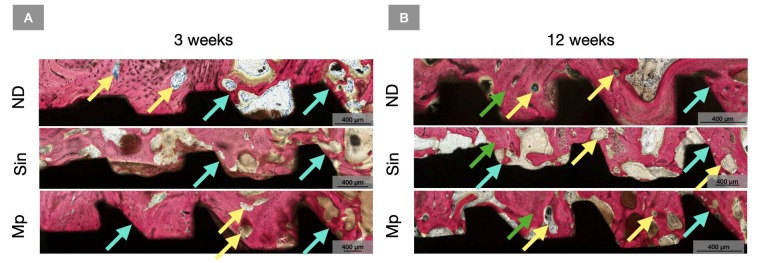
Representative high magnification histomicrographs of ND, Sin, and Mp implants at the crestal aspect at (**A**) 3 weeks and (**B**) 12 weeks. Implants are shown in black, and bone is in red as a result of SVG staining. Yellow arrows point towards regions of bone remodeling, cyan arrows indicate regions of bone-to-implant contact, and green arrows highlight osteocytes within lacunae.

**Figure 7:**
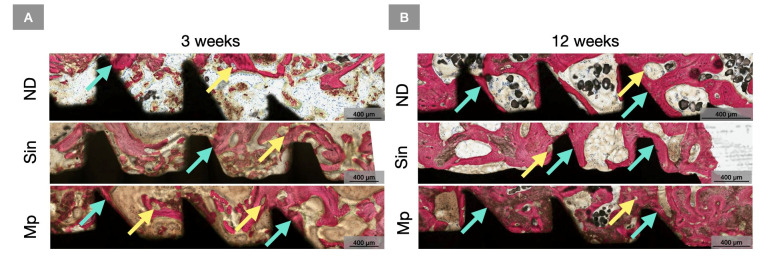
Representative high magnification histomicrographs of ND, Sin, and Mp at the apical aspect at (**A**) 3 weeks and (**B**) 12 weeks. Implants are shown in black, and bone is in red as a result of SVG staining. Yellow arrows point towards regions of bone remodeling and cyan arrows indicate regions of bone-to-implant contact.

BIC was statistically homogeneous among groups at 3 weeks (*p* ≥ 0.468) (Figure 2A). By 12 weeks, a significant difference was observed between ND relative to Sin (*p =* 0.016). Although all implant groups exhibited an overall increase in mean BIC values between 3 and 12 weeks, this change reached statistical significance only in the ND group (*p* = 0.028) (Figure 2B). At both 3 and 12 weeks, Mp demonstrated significantly higher BAFO relative to ND (*p* = 0.015 and *p* = 0.008, respectively) (Figure 3A). Although mean BAFO values increased over time across all groups, these changes were not statistically significant (*p* ≥ 0.099) (Figure 3B). A summary of the BIC and BAFO values are provided in Table 2 and Table 3, respectively. Across all groups, greater cortical bone contact was qualitatively observed along the crestal aspect relative to the apical aspect at both time points. This reflects engagement with the dense cortical plate and transition to deeper trabecular bone (Figure 4 and Figure 5). At 3 weeks in vivo, the healing chambers predominantly consisted of newly forming woven bone in all groups. This was followed by bone maturation at 12 weeks (Figure 6 and Figure 7), as evidenced by the presence of bone remodeling sites and areas of well-organized lamellar structures occupying the healing chambers. Haversian canals and surrounding osteocytes, characteristic of mature bone formation, could be seen in the crestal aspect of all implants at 12 weeks (Figure 7).

## 4. Discussion

Successful osseointegration of endosteal implants depends on early bone formation and long-term stability at the bone–implant interface [9,44,45]. Since BIC reflects direct bone contact, it is often used to assess primary stability [44,46]. At 3 weeks, no significant differences in BIC were observed among the groups which may be attributed to the osteoconductive nature of all implant surfaces [9,44,47]. This is in agreement with findings of Lang et al. who previously demonstrated that the degree of osseointegration around a chemically modified, moderately rough, hydrophilic implant surface was more pronounced at the 2–4 week period relative to a hydrophobic surface [48]. However, differences in BIC between ND and Sin at the 12 week healing time point may be attributed to macrogeometric variations. While both groups presented with V-shaped threads and acid-etched surfaces, ND featured a hybrid design with a section comprising trapezoidal threads. Together, these features have been reported to promote increased compressive engagement and mechanical stability [2,49]. In contrast, the performance of Sin implants may have plateaued due to its HA coating potentially dissolving from the surface [50]. Wennerberg et al. previously demonstrated this using radiolabeled particles, wherein a large portion of HA dissolved from the implant surface by 4 weeks, while only trace amounts could be detected in the liver in a rabbit tibia model by 8 weeks [51]. However, it remains unclear whether the difference in BIC between ND and Sin could be attributed to this phenomenon and warrants further evaluation via inductively coupled ion mass spectroscopy or surface characterization via X-ray photoelectron spectroscopy. On the other hand, ND exhibited higher mean BIC values than Mp at 12 weeks. The literature indicates that changes in the size and shape of healing chambers influences new bone formation within and in proximity to macrogeometric features [35]. The observed trend, although not statistically significant, may be due to the open thread design (decompressing chambers) of the Mp implants, which requires bone growth from both between the threads and within the vertical healing chambers to facilitate direct contact between newly forming hard tissue and implant surface [35].

At both 3 and 12 weeks post-implantation, Mp exhibited significantly higher BAFO relative to ND [7,44]. The vertical decompression chambers in Mp have been shown in previous studies to serve as regions for early vascular invasion, osteoblast and chondrocyte migration, and subsequent bone matrix deposition, thus improving secondary biologic stability [36,52,53]. This process reduces reliance on surrounding cortical compression for implant stability, which is particularly important in trabecular bone (characterized by lower cortical bone engagement) [8,44,54,55]. In contrast, self-tapping helical cutting flutes, such as in the ND implants, are primarily designed to reduce resistance during implant placement as opposed to supporting long-term peri-implant bone formation [7,56]. In comparison, Sin implants resulted in BAFO values that were comparable to both Mp and ND, likely due to overlapping geometric features, including conical connections, V-shaped threads, and a tapered body, that may have collectively moderated differences in bone response.

Differences in surface coatings may also explain differences in BAFO between Mp and ND. For example, Mp implants underwent dual acid etching and final surface modification with NTP. In contrast, the ND implant featured a grit-blasted, acid-etched surface with final hydrophilic conditioning via isotonic sodium chloride (Acqua™) [20]. Both surface modifications have been shown to decrease surface contact angle values (indicative of hydrophilicity and wettability) in the early stages of bone healing, resulting in improved cellular adhesion and attachment [20,57]. However, the higher BAFO in Mp at 12 weeks may suggest that NTP could offer longitudinal benefits towards osseointegration. Several studies have demonstrated the potential of NTP to influence all stages of the bone healing cascade [32,34]. Mechanistically, these effects are not attributable to a single reactive species alone, but instead, a mixture of RONS generated during NTP treatment [24,25,26]. Since the present study did not directly quantify RONS, the relative contribution of each could not be determined, warranting follow-up studies. However, taken together, the combination of vertical healing chambers, dual acid etching, and NTP treatment promoted early vascular infiltration and sustained bone deposition. Beyond these histological benefits, the NTP technique offers distinct clinical advantages including the ability to be used in situ (chair-side) during implant placement, eliminating the need for specialized laboratory facilities in addition to providing robust antibacterial effects [58,59,60].

Despite its translational relevance, this study is not without limitations. While the sheep model provides a cancellous bone environment comparable to that of a human maxilla, differences in bone remodeling rates and mechanical loading conditions may impact the direct applicability of these findings to clinical practice. Furthermore, the three implant systems evaluated in this study differed in both macro- and micromorphological characteristics. Given this, the observed differences in BIC and BAFO cannot be attributed to a single variable in isolation, and the findings should be interpreted as reflecting a combination of effects. Generalizability of these results are, therefore, limited and direct comparisons with other implants should be made with caution. Future studies are needed to extend the observation period beyond 12 weeks and include mechanical testing (i.e., pull-out force or removal torque) to correlate histological and histomorphometric outcomes with biomechanical stability. Moreover, a controlled study design comparing the same implant system with and without the surface coatings (Acqua™, HAnano^®^, and NTP) is required to isolate their independent effects on osseointegration. Additionally, immunohistochemistry could provide further insights into the biological mechanisms responsible for driving these differences in osseointegration in this type IV bone healing environment.

## Figures and Tables

**Table 1:** Summary of the macrogeometric features and surface characteristics of the implants used in this study [20,42,41].

	ND	Sin	Mp
Length × diameter (mm)	10 × 4	10 × 3.8	10 × 4
Shape	Hybrid	Tapered	Tapered
Thread shape	Hybrid (trapezoidal at coronal aspect, V-shaped at apical aspect)	V-shaped	Buttress with vertical decompressing chambers
Grit blasting	Yes	No	Yes
Acid etching	Single acid etching	Dual acid etching	Dual acid etching
Surface coating	Isotonic NaCl (Acqua™)	Nano-hydroxyapatite (HAnano^®^)	Non-thermal plasma (NTP)

**Table 2:** Summary of the BIC values at the 3 and 12 week time points.

Group	Time	Mean (in %)	95% CI
ND	3 weeks	43.261	7.406
	12 weeks	55.075	7.406
Sin	3 weeks	39.651	7.406
	12 weeks	42.267	7.406
Mp	3 weeks	41.994	7.406
	12 weeks	46.985	7.406
**Pairwise Comparisons as a Function of Time**			
Group	Time	Time	*p*-value
ND	3 weeks	12 weeks	0.028
	12 weeks	3 weeks	0.028
Sin	3 weeks	12 weeks	0.614
	12 weeks	3 weeks	0.614
Mp	3 weeks	12 weeks	0.338
	12 weeks	3 weeks	0.338
**Pairwise Comparisons as a Function of Group**			
Time	Group	Group	*p*-value
3 weeks	ND	Sin	0.468
		Mp	0.798
	Sin	ND	0.468
		Mp	0.636
	Mp	ND	0.798
		Sin	0.636
12 weeks	ND	Sin	0.016
		Mp	0.113
	Sin	ND	0.016
		Mp	0.345
	Mp	ND	0.113
		Sin	0.345

**Table 3:** Summary of the BAFO values at the 3 and 12 week time points.

Group	Time	Mean (in %)	95% CI
ND	3 weeks	40.002	7.386
	12 weeks	46.773	7.386
Sin	3 weeks	45.547	7.386
	12 weeks	54.245	7.386
Mp	3 weeks	52.424	7.386
	12 weeks	60.573	7.386
**Pairwise Comparisons as a Function of Time**			
Group	Time	Time	*p*-value
ND	3 weeks	12 weeks	0.195
	12 weeks	3 weeks	0.195
Sin	3 weeks	12 weeks	0.099
	12 weeks	3 weeks	0.099
Mp	3 weeks	12 weeks	0.121
	12 weeks	3 weeks	0.121
**Pairwise Comparisons as a Function of Group**			
Time	Group	Group	*p*-value
3 weeks	ND	Sin	0.249
		Mp	0.015
	Sin	ND	0.249
		Mp	0.157
	Mp	ND	0.015
		Sin	0.157
12 weeks	ND	Sin	0.126
		Mp	0.008
	Sin	ND	0.126
		Mp	0.191
	Mp	ND	0.008
		Sin	0.191

## Data Availability

The original contributions presented in this study are included in the article. Further inquiries can be directed to the corresponding authors.
